# Awful Mortality on Board H. M. Steamer Eclair

**Published:** 1846-01

**Authors:** 


*Awful Mortality on boar.l H. M. Steamer Eclair.*—The Eclair steam-sloop, Commander Walter G. B. Estcourt, arrived at the Motherbank on Monday, with the yellow flag, with a black ball in its centre, flying from her mainmast head, emblematic of death on board.

She has been so awfully visited with sickness since she has been on the coast of Africa that she has been sent home. No less than sixty-five have died in the vessel, and others were dying hourly. The Eclair was sent off the station by the orders of Commander C. H. M. Buckle of the Growler steam-sloop; prior to which, however, on the disease manifesting itself in the deadly form it assumed, a commission of three surgeons sat upon the case to decide what was best to be done under the alarming circumstances, when they came to the unanimous resolution of sending the Eclair off the station and home. The names of the officers dead are Commander Estcourt; the surgeon, Mr. John Maconchy; the paymaster and purser, Mr. Thomas R. Hallett; the assistant surgeon, Mr. Charles Hartman; the clerk, Mr. Cleland Mill; naval cadet, Mr. Symons; master's assistant, Mr. Goman. The Eclair was only commissioned last August twelve months, and is a new vessel (first named the Lucifer,) of 350 horse-power. No one is allowed to go on board of or to leave the vessel. The Echo tug has, however, towed down a lighter with 30 tons of coal, stores, &c., on board, for the use of the vessel. This stock will be moored to a buoy, and left for the Eclair's survivors to take on board. After it is shipped she will leave for Standgate creek, near Sheerness, and there ride out forty days or more after clean bills of health have been received from her.—*London Examiner.*

